# Expression and cytosolic assembly of the S-layer fusion protein mSbsC-EGFP in eukaryotic cells

**DOI:** 10.1186/1475-2859-4-28

**Published:** 2005-10-04

**Authors:** Andreas Blecha, Kristof Zarschler, Klaas A Sjollema, Marten Veenhuis, Gerhard Rödel

**Affiliations:** 1 Institut für Genetik, Technische Universität Dresden, D-01062 Dresden, Germany.; 2 Eukaryotic Microbiology, Groningen Biomolecular Sciences and Biotechnology Institute (GBB), University of Groningen, PO Box 14, NL-9750 AA Haren, The Netherlands.

## Abstract

**Background:**

Native as well as recombinant bacterial cell surface layer (S-layer) protein of *Geobacillus (G.) stearothermophilus *ATCC 12980 assembles to supramolecular structures with an oblique symmetry. Upon expression in *E. coli*, S-layer self assembly products are formed in the cytosol. We tested the expression and assembly of a fusion protein, consisting of the mature part (aa 31–1099) of the S-layer protein and EGFP (enhanced green fluorescent protein), in eukaryotic host cells, the yeast *Saccharomyces cerevisiae *and human HeLa cells.

**Results:**

Upon expression in *E. coli *the recombinant mSbsC-EGFP fusion protein was recovered from the insoluble fraction. After denaturation by Guanidine (Gua)-HCl treatment and subsequent dialysis the fusion protein assembled in solution and yielded green fluorescent cylindric structures with regular symmetry comparable to that of the authentic SbsC. For expression in the eukaryotic host *Saccharomyces *(*S*.) *cerevisiae *mSbsC-EGFP was cloned in a multi-copy expression vector bearing the strong constitutive *GPD*1 (glyceraldehyde-3-phosophate-dehydrogenase) promoter. The respective yeast transfomants were only slightly impaired in growth and exhibited a needle-like green fluorescent pattern. Transmission electron microscopy (TEM) studies revealed the presence of closely packed cylindrical structures in the cytosol with regular symmetry comparable to those obtained after *in vitro *recrystallization. Similar structures are observed in HeLa cells expressing mSbsC-EGFP from the Cytomegalovirus (CMV IE) promoter.

**Conclusion:**

The mSbsC-EGFP fusion protein is stably expressed both in the yeast, *Saccharomyces cerevisiae*, and in HeLa cells. Recombinant mSbsC-EGFP combines properties of both fusion partners: it assembles both *in vitro *and *in vivo *to cylindrical structures that show an intensive green fluorescence. Fusion of proteins to S-layer proteins may be a useful tool for high level expression in yeast and HeLa cells of otherwise instable proteins in their native conformation. In addition the self assembly properties of the fusion proteins allow their simple purification. Moreover the binding properties of the S-layer part can be used to immobilize the fusion proteins to various surfaces. Arrays of highly ordered and densely structured proteins either immobilized on surfaces or within living cells may be advantageous over the respective soluble variants with respect to stability and their potential interference with cellular metabolism.

## Background

Bacterial cell surface layers (S-layer) as the outermost cell envelope components are a common feature of many bacteria and archaea species (for review see [1,2]). With few exceptions S-layers consist of a single species of subunits that occasionally is posttranslationally modified by phosphorylation [3], or glycosylation [4,5]. S-layer monomers assemble to two-dimensional highly porous arrays with either oblique, square or hexagonal symmetry. The interactions between the S-layer subunits as well as between the S-layer and the supporting envelope can be disrupted in a reversible manner by cation substitution or high concentrations of chaotropic agents [6]. Upon removal of the denaturing agent the isolated S-layer subunits assemble *in vitro *into regular arrays exhibiting structural features of the authentic cell surface layer. Contrary to the situation *in vivo*, the *in vitro *self-assembly process of S-layer proteins in solution can also result in double layer sheets or in tube-like structures [7].

S-layers have been recognized as important structures for biotechnological applications [8]. However, large-scale preparation of S-layers from the authentic organisms is sometimes limited. For example, some bacterial strains have been reported to loose their ability to produce S-layers under laboratory conditions [9]. Due to alterations in the cultivation conditions expression of truncated forms of the S-layer protein may result in the loss of S-layer sheets [10]. To circumvent such difficulties recombinant S-layer proteins have been heterologously produced in prokaryotic systems, like *E. coli*, *Bacillus subtilis*, *Lactobacillus casei *[9], or *Lactococcus lactis *[11]. For example, high level expression of the S-layer protein SbsC from *Geobacillus stearothermophilus *ATCC 12980 has been reported in *E. coli *[12]. SbsC possesses an N-terminal secretion signal of 30 amino acids (aa) that is cleaved off during secretion, a secondary cell wall polymer (SCWP)-binding domain (aa 31–258), and a central portion responsible for formation of lattice symmetry and self-assembly [12]. Upon expression in *E. coli*, the mature S-layer protein mSbsC_(31–1099) _with an apparent molecular mass of 112 kDa forms monolayer cylinders and spirally wound sheets-like structures in the cytosol that can be recovered from the insoluble fraction upon cell lysis. Neither deletion of the C-terminal 179 aa (rSbsC_(31–920)_) [7] nor fusion of this truncated form with major birch pollen antigen Bet v1 (rSbsC_(31–920)_-Betv1) interferes with the assembly process and the oblique lattice symmetry. Interestingly, the Bet v1 epitope was accessible to antibodies demonstrating that the fused portion is protruding from the respective assembly structure [13]. Contrary to the situation of SbsC, recombinant S-layer protein SbpA from *B. sphaericus *CCM 2177 and its derivatives fail to assemble in the *E. coli *cytosol, but form insoluble inclusion bodies [14].

In the present study we addressed the question whether the cytosol of eukaryotic host cells can provide a suitable environment for the formation of S-layer self assembly products, despite the presence of numerous chaperons and proteases. Assembly of S-layer fusion proteins could provide a simple purification scheme for soluble proteins, especially if the formation of their native structures depends on the conditions of the eukaryotic cytosol. In addition, such fusion proteins could stabilize proteolytically sensitive proteins and thus increase their yield.

We used the yeast *Saccharomyces cerevisiae *and the HeLa cells as eukaryotic model organisms for expression of a bifunctional S-layer fusion protein, consisting of mature SbsC (aa 31–1099) and the enhanced green fluorescent protein (EGFP).

## Results

### Expression of mSbsC-EGFP in *E. coli*

**Figure 1:**
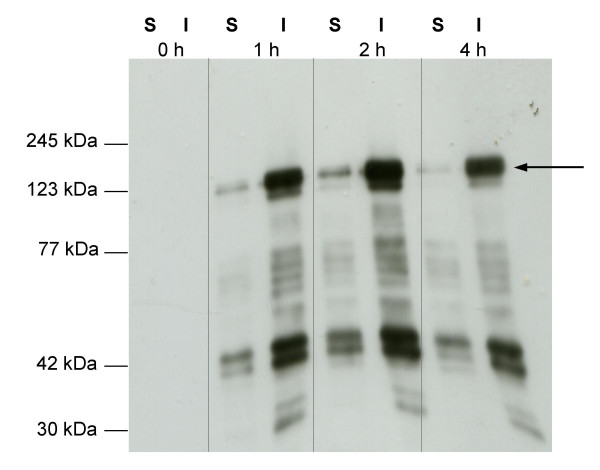
**Expression of mSbsC-EGFP in *E. coli*. **Expression of mSbsC-EGFP in *E. coli *transformants was induced by addition of IPTG as described in Material and methods. Immediately before induction (0 h), 1 h, 2 h and 4 h after induction samples of the cells were lysed and the soluble (S) and insoluble (I) fractions were prepared. 10 μg of each fraction were subjected to Western blot analysis with anti-GFP antibody. Sizes of marker proteins (Roti^®^-Mark prestained, Carl Roth GmbH; M) are indicated on the left hand side. Arrow indicates the full-size protein of 140 kDa.

The 3207 bp open reading frame (ORF) encoding mSbsC_(31–1099) _was fused with the 720 bp ORF of EGFP and cloned into pET17b as described in Methods. Both ORFs are separated by a two-aa-linker (Leu-Glu) that was introduced by the non-template-encoded *Xho*I site (CTCGAG). *E. coli *BL21(DE3), transformed with pET17b-mSbsC-EGFP, were cultivated at 30°C and 37°C and analysed by fluorescence microscopy. Expression at 30°C resulted in an enhanced fluorescence emission, indicating a higher yield of correctly folded EGFP [14]. 0, 1, 2, and 4 hours after induction by IPTG cell lysates were tested for the presence of the fusion protein by Western blot analysis with anti-GFP antibody. Already after 1 h the mSbsC-EGFP-fusion protein, with an expected molecular weight of 139 kDa, could be detected (Fig. 1). The protein was recovered from the pellet fraction, indicating the formation of inclusion bodies or of assembly products as recently reported in the case of mature SbsC [7]. In addition to the predominant 139 kDa band a number of low molecular weight protein bands are detected by the anti-GFP antibody, that likely reflect degradation products, although internal start of translation cannot be excluded.

**Figure 2:**
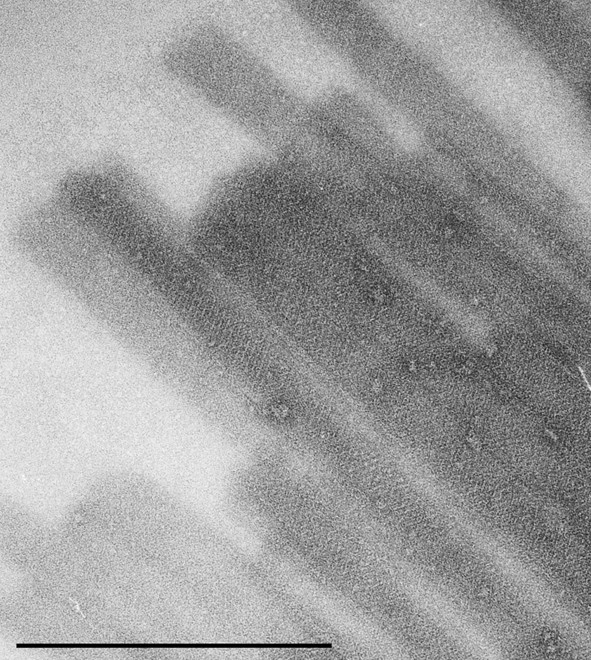
**TEM analysis of recrystallized recombinant mSbsC-EGFP. **Recombinant mSbsC-EGFP was isolated from *E. coli *transformants, denatured with 5 M Gua-HCl, dialysed against dH_2_O, and the assembled structures were subjected to TEM-analysis. The cylindrical structures with a diameter of approximately 60 nm exhibit regular symmetry. bar = 1μm

Pure preparations of mSbsC-EGFP were obtained by FPLC as described in Methods. REM analysis of dialysed FPLC fractions of the purified full-size mSbsC-EGFP revealed predominantly tube-like structures. According to TEM analysis these tubes exhibit a regular symmetry (Fig. 2). The cylindrical structures had an average length of 10–20 μm and a diameter of approximately 60 nm. This partly resembles the situation of mSbsC which has been described to assemble into sheets and cylindric structures (albeit with a diameter of 70–110 nm) [7].

When recrystallization was performed in the presence of *G. stearothermophilus *wild type strain ATCC 12980 devoid of its native S-layer due to Gua-HCl treatment, cells were covered with a green fluorescent layer indicating binding to the SCWP (data not shown). The same result was obtained with other *G. stearothermophilus *strains (DSM 297, DSM 13240 and DSM 1550), but not with the Gua-HCl treated yeast strain BY4741.

### Expression of mSbsC-EGFP in *S. cerevisiae*

**Figure 3:**
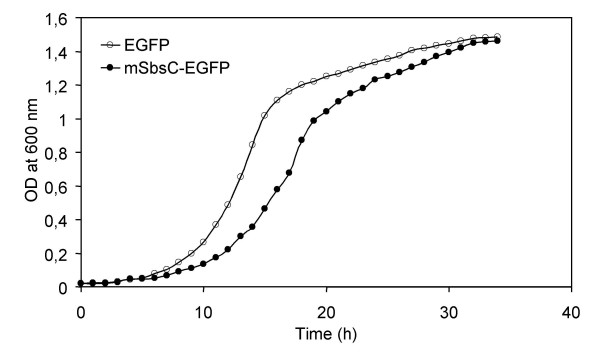
**Growth curves of EGFP and mSbsC-EGFP expressing yeast transformants. **Batch cultures of *S. cerevisiae *strain BY4741 expressing EGFP (open circles) or mSbsC-EGFP (filled circles) were inoculated in selective minimal medium with an OD_600 _of 0,02 and grown for 30 h at 30°C. Growth was followed by the increase of OD_600_.

**Figure 4:**
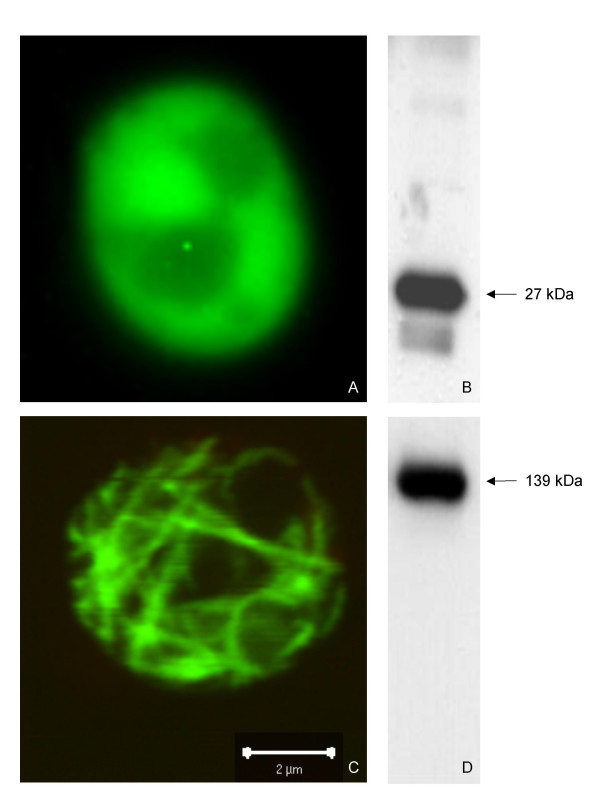
**Fluorescence microscopy of EGFP and mSbsC-EGFP expressing yeast transformants. **Yeast cells expressing EGFP (A) or mSbsC-EGFP (C) were cultivated in selective minimal medium to exponential phase and analysed by fluorescence microscopy. Westernblot analysis of whole cell extract using anti-GFP antibodies reveal the presence of either EGFP (B) or mSbsC-EGFP (D). bar = 2 μm

For expression in a eukaryotic system p426-GPD-mSbsC-EGFP was transformed into the *S. cerevisiae *strain BY4741. As a control BY4741 was transformed with p426-GPD-EGFP. Expression of the S-layer fusion protein has only a very moderate effect on growth of the transformants (Fig. 3). Despite of a slightly prolonged lag phase and a marginally elongated doubling time, cell densities in the stationary phase are identical. Western blot analysis of the 20,000 × g pellet from whole cell lysates with anti-GFP antibody showed a strong signal by a distinct protein band whose size is identical with that of mSbsC-EGFP expressed in *E. coli *(Fig. 4C). Contrary to the situation in *E. coli *no further signals were detected, indicating that expression mSbsC-EGFP in yeast is not accompanied by any obvious degradation. Fluorescence microscopy revealed that EGFP-expressing cells show a homogeneous distribution of green fluorescence (Fig. 4A), while mSbsC-EGFP expression results in the formation of green fluorescent needle-like structures in the cytosol (Fig. 4C). Contrary to the situation of EGFP-expressing cells which all exhibit a similar fluorescence intensity, yeast cells expressing the fusion protein vary widely in their green fluorescence from low to extremely strong signals.

### TEM analysis of recombinant mSbsC-EGFP structures in yeast

**Figure 5:**
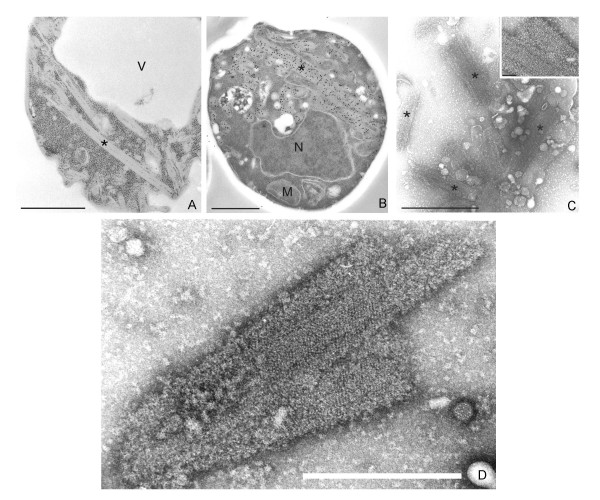
**TEM-analysis of ultra-thin section of a yeast transformant expressing mSbsC-EGFP. **(A) mSbsC-EGFP expressing yeast spheroplast showing rod like structures (*). (B) Immunocytochemistry of mSbsC-EGFP expressing yeast cell using antibodies against GFP. (C) Negative staining of osmotically shocked yeast protoplasts expressing mSbsC-EGFP showing *in vivo *formed assembly products with similar structures (*) as in Fig 2, the inset and image (D) show a higher magnification of these structures, a lattice is discernible. Key: M-mitochondrion, N-nucleus, V-vacuole. The bar represents 1 μm, for the inset 100 nm.

TEM analysis of ultrathin sections of whole cells reveals tube-like self assembly products, that are exclusively located in the cytosol and neither associated with organelles, the cytoskeleton, or the plasma membrane (Fig. 5A). To verify the identity of these structures as the product of mSbsC-EGFP self assembly, anti-GFP antibody in combination with a secondary colloidal gold-conjugated antibody were used for detection in thin-sectioned protoplasts. Figure 5B shows that the densely packed protein crystals are selectively immunogold-labeled with primary anti-GFP antibody, thus confirming their identity as the recombinant S-layer fusion protein. TEM analysis of gently disrupted protoplasts revealed cylindrical, closely packed structures (Fig. 5C) exhibiting a regular symmetry (insert, for a more detailed image see 5D). These results show that mSbsC-EGFP monomers possess the intrinsic ability to form highly ordered assembly structures comparable to those observed after *in vitro *crystallization of mSbsC-EGFP (see Fig. 2).

### Expression of mSbsC-EGFP in HeLa-cells

The ORFs of mSbsC and EGFP were fused in-frame within the vector pEGFP-N1. HeLa cells were liposome-transfected with the resulting plasmid pmSbsC-EGFP-N1. 16 h after transfection adherent HeLa cells exhibit green fluorescent filamentous structures. The fluorescence was exclusively restricted to these structures, other intracellular areas were competely devoid of green fluorescence. Obviously the fluorescent structures result from self assembly of mSbsC-EGFP within the cytosol. The morphology of these structures is very similar to that of the assembly products formed in mSbsC-EGFP producing yeast cells.

**Figure 6:**
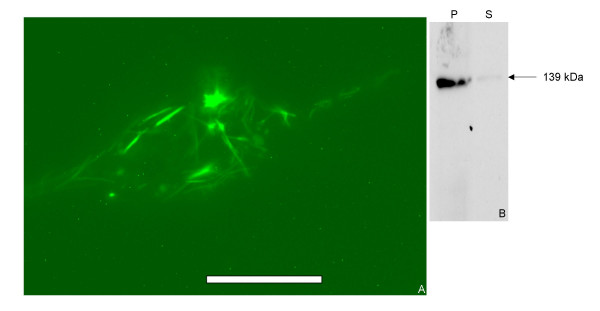
**Fluoresence microscopy and Western blot analysis of HeLa cells expressing mSbsC-EGFP. **(A) mSbsC-EGFP expressing HeLa cells 16 h after liposome transfection. Adherent cells show a filament-like green fluorescent pattern that resembles the structures obtained in yeast cells expressing mSbsC-EGFP. (B) Western blot analysis of lysate from mSbsC-EGFP expressing HeLa cells. Lysed cells were centrifuged at 20,000 × g and the resulting supernatant (S) and pellet (P) fractions were analysed by SDS-PAGE and Western blot with anti-GFP antibody. The mSbsC-EGFP fusion protein is mainly present in the insoluble fraction.

Transfected HeLa-cells were mechanically lysed with a dounce homogenizer, separated in a soluble and a pellet fraction by high speed centrifugation (20,000 × g), and the fractions analysed in a Western blot using anti-GFP antibodies. As shown in Figure 6, mSbsC-EGFP can be detected as an abundant protein band in the range of 139 kDa in the pellet fraction. Similar to the situation in yeast transformants we did not observe degradation products, indicating low or absent proteolytic activities.

## Discussion

Despite of their potential for biotechnological application only few S-layers genes have been identified and were used for genetic modification and recombinant production. Usually prokaryotic systems like *Lactobacillus casei*, *B. subtilis*, *E. coli *(for review see: [9]) and *Lactococcus lactis *[11] were used for heterologous expression.

Our report describes for the first time the expression of a S-layer fusion protein in eukaryotic host cells. Because of the ability of mSbsC to form sheet-like and cylindrical structures in the cytosol of *E. coli *[7], we decided to use this S-layer protein for fusion with EGFP. Expression was first investigated in the yeast *S. cerevisiae*, one of the best-studied eukaryotic organism. We observed no alterations of the cell shape and only a slightly reduced vegetative growth, possibly reflecting the high energy demand due to the constitutive high level synthesis of 139 kDa mSbsC-EGFP. Western blot analysis of insoluble material with anti-GFP antibody gave no indication for N-terminal proteolytic degradation of the recombinant protein as observed in *E. coli*. Possibly the organization of mSbsC-EGFP in assembled structures confers protection from ubiquitin-mediated degradation in yeast. In respect to protein stability *S. cerevisiae *seems to be a superior expression system. Fluorescence microscopy revealed that yeast transformants exhibited an intense green fluorescence indicating proper folding of the EGFP part within mSbsC-EGFP. In line with the formation of self-assembly structures in the cytosol, the fluorescence pattern differs significantly from that of EGFP-expressing yeast cells. TEM studies revealed the presence of closely packed cylindrical structures with regular symmetry comparable with those obtained after *in vitro *recrystallization. Our study demonstrates that the assembly process of SbsC-EGFP is not affected by the presence of eukaryote-specific cytosolic chaperons, like members of the CCT family [15].

Recently it was reported that some S-layer monomers assemble on hydrophobic surfaces. S-layer proteins of the strains *G. stearothermophilus *PV72/p2 and NRS 2004/3a form protein monolayers on substrates like silicon wafers with a native oxide layer [16]. The self-assembly structures of SbsC-fusion proteins in yeast are not associated with the inner surface of the plasma membrane or cellular organelles despite their hydrophobic membranes. Obviously the hydrophobic membrane patches do not act in anchoring assembled S-layer structures.

As in yeast cells, mSbsC-EGFP monomers self assemble into filamentous structures within the cytosol of HeLa cells that are neither associated with subcellular structures nor affect the cell shape. We did not observe necrotic or apoptotic events in the transfected cells that expressed mSbsC-EGFP (data not shown). The detection of a single band of the expected molecular weight in Western blot analysis with anti-GFP antibodies indicates that the recombinant protein is stable and not subjected to N-terminal degradation.

A positively charged N-terminal domain of S-layer proteins directly interacts via electrostatic forces with the negatively charged SCWP whose chemical composition is identical in *G. stearothermophilus *wild-type strains [17]. In our study we show that fusion of EGFP to the mSbsC, resulting in an extension of the S-layer protein by ~240 aa, does not interfere with the binding.

## Conclusion

Our results show that the mSbsC-EGFP fusion protein is efficiently synthesized, and self-assembles under the physiological conditions of the cytosol of eukaryotic cells. Both in yeast and human cells cytosolic accumulation of S-layer self assembly products is not accompanied by proteolytic degradation. The green fluorescence of mSbsC-EGFP suggests that both *in vitro *and *in vivo *folding of both protein portions within the fusion protein is independent, and not affected by each other. This observation opens the possibility to fuse other proteins, *e.g*. enzymes, to the S-layer part in order to obtain functional fusion constructs in a densely packed and stable structure.

Fusion of otherwise instable proteins to S-layer proteins may be a useful tool for their high level expression in eukaryotic cells. The self assembly properties of the fusion proteins can be exploited to purify them by simple centrifugation steps from cell lysate. By engineering a suitable protease cleavage site between the S-layer and the fused protein part, incubation of the assembly products with the respective proteases could allow separation and subsequent purification of the protein of interest. Arrays of highly ordered and densely structured S-layer fusion proteins can efficiently be immobilized on a variety of surfaces, either directly or upon treating with SCWP.

Besides technical applications, an ordered aggregation of S-layer fusion proteins within living cells may offer novel strategies for cell manipulation. For example, if expression of proteins would interfere with cellular functions, e.g. as a result of intracellular transport, fusion to an S-layer protein permits their retention in the cytosol. "*In vivo *affinity chromatography" may be another potential field for applying S-layer fusion proteins. Enrichment of soluble biomolecules within living cells may be achieved by assembly structures consisting of fusion proteins between the respective receptor and a suitable S-layer protein.

## Methods

### Strains

*Geobacillus (G.) stearothermophilus *ATCC 12980

*G. stearothermophilus *DSM 1550 (German Collection of Microorganisms and Cell Cultures (DSMZ) GmbH, Braunschweig, Germany)

*G. stearothermophilus *DSM 13240 (DSMZ)

*G. stearothermophilus *DSM 297 (DSMZ)

*Escherichia (E.) coli *DH5á (BRL)

*E. coli *BL21(DE3) (Invitrogen)

*Saccharomyces *(*S*.) *cerevisiae *BY4741 (EUROSCARF)

HeLa cells (kind gift of Frank Pfennig, Institut für Zoologie, Technische Universität Dresden, Germany)

### Primers

#1: 5' TATATATACATATGGCAACGGACGTGGCGACGGTC 3'

#2: 5' TATATATACTCGAGCGATGCTGATTTTGTACCAATTTG 3'

#3: 5' TATATATACTCGAGATGGTGAGCAAGGGCGAGGAG 3'

#4: 5' TATATATAGCTCAGCTTACTTGTACAGCTCGTCCATGC 3'

#5: 5' TATATATAGGATCCATGGCAACGGACGTGGCGACGGTC 3'

#6: 5' TATATATAGGTACCTCACTATTACTTGTACAGCTCGTCCATGC 3'

#7: 5' TATATATACTCGAGATGGCAACGGACGTGGCG 3'#8: 5' TATATATACCGCGGCGATGCTGATTTTGTACCAATTTG 3'

### Cultivation of *G. stearothermophilus *strains, isolation and recrystallization of S-layer proteins

Batch cultures of the *G. stearothermophilus *strains were grown in tryptone-enriched LB-medium at 55°C (strain DSM 13240 at 65°C) to an OD_600 _of 0,6. Surface proteins attached to the cell wall layer via noncovalent forces were removed by Guanidine-HCl (Gua-HCl) treatment of cells from a 500 ml culture. Cells were washed 3 times in distilled water, suspended in 10 ml of 5 M Gua-HCl and incubated for 30 min on ice with occasional mixing. Cells devoid of their S-layers were recovered by centrifugation at 8,000 × g and washed twice with 5 M Gua-HCl. For preparation of S-layer proteins the supernatant obtained after centrifugation at 40,000 × g was dialyzed against distilled water for 18 h.

### Preparation of genomic DNA and DNA-cloning

Genomic DNA was isolated with the QIAGEN Genomic-tip 100 kit according to the manufacturer's instruction. Plasmid DNA was prepared with the "Wizard^® ^SV Gel and PCR Claen-up system kit" (Promega).

### Cloning in pET17b

DNA encoding mSbsC_(31–1099) _was PCR-amplified with primers #1 and #2, using total DNA of *G. stearothermophilus *ATCC 12980 as a template. An ATG initiation codon was introduced 5'to the open reading frame by primer #1. The PCR-product was cut with *Nde*I and *Xho*I and ligated with plasmid pET17b (Novagen) to yield plasmid pET17b-mSbsC_oT.

The *Xho*I and *Bpu*1102I sites of pET17b-mSbsC_oT were used to insert the EGFP reading frame, which was PCR-amplified from vector EGFP-N1 (Clontech) with primers #3 and #4. Primer #3 introduced a 5'-flanking *Xho*I-site in the PCR product, while primer #4 generated a 3'-flanking *Bpu*1102I-site and introduced a TAA termination codon. The PCR-product was cut with *Xho*I and *Bpu*1102I and ligated with pET17b-mSbsC_oT to yield plasmid pET17b-mSbsC_oT-EGFP.

### Cloning in p426-GPD

A DNA fragment encoding mSbsC was PCR-amplified as described above with primers #5 and #2, cut with *Bam*HI and *Xho*I, and ligated with the *S. cerevisiae *expression vector p426-GPD, which bears the strong *GPD *promoter [18]. The resulting plasmid p426-GPD-mSbsC_oT was used for *in frame *fusion the EGFP orf, which was amplified with primers #3 and #6. The respective PCR product harbours a 5' *Xho*I site and at the 3'-end three consecutive termination codons followed by a *Kpn*I restriction site. Thereby plasmid p426-GPD-mSbsC_oT-EGFP was created.

Plasmid p426-GPD-EGFP for expression of EGFP in yeast was created by ligation of the PCR-fragment encoding the EGFP orf into p426-GPD *via *the flanking *Xho*I and *Kpn*I sites. Plasmids p426-GPD-mSbsC_oT-EGFP and p426-GPD-EGFP were propagated in *E. coli *DH5α and transformed into the *S. cerevisiae *wild type strain BY4741.

### Cloning in pEGFP-N1

The reading frame coding for mSbsC was amplified with primers #7 and #8 and subsequently integrated within the *Xho*I and *Sac*II restriction sites in the vector pEGFP-N1 (Clontech) as an *in frame *fusion with the EGFP-ORF.

The resulting plasmid pmSbsC-EGFP-N1 was propagated in *E. coli *DH5α and transfected into HeLa cells.

### Transfection of HeLa cells

For transient transfection with plasmid pmSbsC-EGFP-N1, 2,5 × 10^5 ^cells were plated on chamber slides (Nunc) and incubated after one day with TFX™-20 liposome reagent (Promega) and 3 μg DNA for 1 h. Subsequently DMEM with high glucose and 10 % (v/v) fetal calf serum (PAA laboratories) was supplemented. After over night cultivation transfected cells were analysed by Western blot and fluorescence microscopy.

### Isolation and purification of recombinant S-layer proteins from *E. coli*

*E. coli *BL21(DE3) was used as a prokaryotic host for the expression of mSbsC-EGFP. *E. coli *transformants bearing pET17b-mSbsC-EGFP were cultivated in LB medium containing 100 mg/l ampicillin at 30°C to an OD_600 _of 0,4. S-layer synthesis was initiated by adding IPTG to a final concentration of 0,5 mM.

8–16 h after induction cells obtained from a 500 ml culture were harvested by centrifugation at 10,000 × g for 10 min; 4°C. The cell pellet was washed 3 times with ice-cold distilled water and resuspended in 10 ml Tris-buffer (10 mM Tris, pH 7,5; 1 mM AEBSF hydrochloride (AppliChem)) containing 1% (v/v) Triton X-100. After disruption of cells by French press (25,000 PSI pressure, at 4°C), soluble and insoluble fractions were separated by centrifugation at 20,000 × g for 30 min at 4°C. Nucleic acids were removed from the pellet fraction by treatment with DNase I (1 mg/ml) and RNase A (1 mg/ml) in Tris-buffer. After three washing steps with Tris-buffer the insoluble material was denatured in 5 ml 5 M Gua-HCl (in 10 mM Tris pH 7,5). The suspension was stirred at room temperature (RT) for 30 min and centrifuged for 30 min at 40,000 × g (10°C). Renatured S-layer proteins were obtained by dialyzing the supernatant twice against 5 l of distilled water or Tris-buffer over night at 4–8°C using dialysis tubes with an exclusion size of 14 kDa.

For further purification the suspension containing renatured S-layer proteins was centrifuged at 12,000 × g for 30 min, the pellet washed 3 times with distilled water or Tris-buffer and dissolved in 3 M Gua-HCl in Tris-buffer. After a final centrifugation step (40,000 × g; 30 min) the clear supernatant was subjected to FPLC using a Sephacryl™ S-300 column (Pharmacia Biotech). Size exclusion chromatography was performed under denaturing conditions (3 M Gua-HCl in Tris-buffer) at RT with a flow rate of 0,5 ml/min. Fractions containing mSbsC-EGFP were dialyzed against dH_2_O over night and used for further analysis.

### Isolation and purification of recombinant S-layer proteins from *S. cerevisiae*

Constitutive expression of S-layer protein coding genes was obtained by growing transformants of *S. cerevisiae *strain BY4741 bearing plasmid p426-GPD-mSbsC-EGFP in YNB medium (Invitrogen) supplemented with Leu, His, Met and glucose as carbon source. 500 ml of an exponential culture (OD_600 _= 1) were centrifuged at 5,000 × g for 3 min and 20°C. Cells were washed 3 times with Tris-buffer and subsequently lysed by osmotic swelling of protoplasts obtained by treatment with zymolyase-20T (ICN) as described [19]. S-layer self assembly products were pelleted from the suspension by centrifugation at 20,000 × g.

### Isolation and purification of recombinant S-layer proteins from HeLa cells

4 × 10^6 ^HeLa cells were transiently transfected with pmSbsC-EGFP-N1. After cultivation for 16 h cells were harvested by trypsin treatment followed by three washing steps with 1 × PBS w/o Ca^2+^, Mg^2+ ^(PAA-Laboratories). Cell lysis was performed by 30 strokes in a dounce homogenizer in the presence of 0,1 % (v/v) Triton X-100, 1 mM AEBSF and Pi-cocktail (protease inhibitor mix; Roche). The cell lysate was centrifuged at 20,000 × g, and the resulting supernatant and pellet fractions were analysed in a Western blot.

### Protein analysis

Sample preparation and SDS-polyacrylamide gelelectrophoresis were carried out as described by Laemmli [20]. Unless otherwise indicated 10 μg of protein were separated on 7,5% (w/v) acrylamide gels. For Westernblot analysis proteins were transferred onto a PVDF membrane (Millipore) and probed with monoclonal mouse antibody directed against the GFP epitope (Boehringer Mannheim). Detection of bound antibodies was performed with horseradish peroxidase (HRP)-conjugated secondary antibodies and the ECL-Plus Kit (Amersham Pharmacia Biotech). As positive control for immunreactivity of anti-GFP antibodies whole cell extract of EGFP expressing *S. cerevisiae *transformants was used (Fig. 4B).

### Electron microscopy

Whole yeast cells and spheroplasts were fixed and prepared for electron microscopy as described by Waterham *et al*. [21]. Immunolabeling was performed on ultrathin sections of Unicryl-embedded cells using polyclonal rabbit anti-GFP antibodies and 15 nm colloidal gold-conjugated goat anti-rabbit secondary antibodies (Amersham). Negative staining of osmotically swollen protoplasts and FPLC-purified recrystallized mSbsC-EGFP was done with 3% (w/v) ammonium-molybdate, pH 7,2 and examined in a Philips CM10 Transmission Electron Microscope.

### Miscellaneous procedures

Standard DNA techniques were as described [22]. Yeast cells were transformed by the lithium acetate method [23]. GENOMED™ columns were used for isolation of DNA fragments from agarose gels. The correct sequence of all constructs was confirmed by DNA sequencing with the dideoxy-chain termination method [24] using 5'-IRD800 labelled primers (MWG-BIOTECH). A Thermo Sequenase fluorescent labelled primer cycle sequencing kit with 7-deaza-dGTP (Amersham) was employed for sequencing with the LI-COR DNA sequencer 4000 (MWG-BIOTECH). Protein concentrations were determined by the Lowry method (BioRad).

## Authors' contributions

AB carried out the molecular genetic studies, performed protein purification and drafted the manuscript.

KZ performed part of the DNA cloning and sequencing and carried out the transfection studies of HeLa cells.

KAS performed the immunogold labeling of yeast protoplasts and the TEM analysis. He provided the TEM images for publication.

MV contributed to the design of the study and conducted the electron microscopy analysis.

GR conceived of the study, participated in its design and coordination, and participated in drafting of the manuscript.

All authors read and approved the final manuscript.
